# Molecular and Functional Characterization of Odorant Binding Protein 7 From the Oriental Fruit Moth *Grapholita molesta* (Busck) (Lepidoptera: Tortricidae)

**DOI:** 10.3389/fphys.2018.01762

**Published:** 2018-12-10

**Authors:** Xiu-Lin Chen, Guang-Wei Li, Xiang-Li Xu, Jun-Xiang Wu

**Affiliations:** ^1^Key Laboratory of Plant Protection Resources and Pest Management (Northwest A&F University), Ministry of Education, Yangling, China; ^2^Shaanxi Province Key Laboratory of Jujube, College of Life Science, Yan' an University, Yan'an, China

**Keywords:** *Grapholita molesta*, odorant binding protein, olfaction, fluorescence binding assay, tissue expression

## Abstract

Odorant-binding proteins (OBPs) are widely and abundantly distributed in the insect sensillar lymph and are essential for insect olfactory processes. The OBPs can capture and transfer odor molecules across the sensillum lymph to odorant receptors and trigger the signal transduction pathway. In this study, a putative OBP gene, *GmolOBP*7, was cloned using specific-primers, based on the annotated unigene which forms the antennal transcriptome of *Grapholita molesta*. Real-time PCR (qRT-PCR) analysis revealed that *GmolOBP*7 was highly expressed in the wings of males and the antennae of both male and female adult moths, while low levels were expressed in other tissues. The recombinant GmolOBP7 (rGmolOBP7) was successfully expressed and purified via Ni-ion affinity chromatography. The results of binding assays revealed that rGmolOBP7 exhibited a high binding affinity to the minor sex pheromone 1-dodecanol containing *K*_*i*_ of 7.48 μM and had high binding capacities to the host-plant volatiles, such as pear ester, lauraldehyde and α-ocimene. RNA-interference experiments were performed to further assess the function of GmolOBP7. qRT-PCR showed that the levels of mRNA transcripts significantly declined in 1 and 2 day old male and female moths, treated with *GmolOBP*7 dsRNA, compared with non-injection controls. The EAG responses of dsRNA-injected males and females to pear ester, as well as the EAG responses of dsRNA-injected males to 1-dodecanol, were significantly reduced compared to the GFP-dsRNA-injected and non-injected controls. We therefore infer that GmolOBP7 has a dual function in the perception and recognition of the host-plant volatiles and sex pheromones.

## Introduction

The sophisticated olfactory system plays an essential role in an insect's survival and reproduction. Adult insects greatly depend on olfactory cues to locate mates and optimal host plants and avoiding predators (Takken and Knols, 1999; Leal, 2013; Suh et al., 2015). In the early events of olfactory processing, airborne chemical signals must pass through the aqueous barrier of the sensillum lymph, surrounding the dendrites of the olfactory receptor neuron (ORNs) cells (Li et al., 2015). Odorant-binding proteins (OBPs), a kind of transport protein, can selectively bind and carry hydrophobic odorants across the sensillum lymph to odorant receptors (ORs) and trigger the signal transduction pathway (Pelosi et al., 2005). The converted electrophysiological signals are then sequentially processed in the antennal lobes, mushroom bodies and central nervous area, to induce a behavioral response in specific semiochemicals of insects (Feng and Prestwich, 1997; Helfrich-Förster, 2000; Hallem et al., 2006; Pelosi et al., 2006; Leal, 2013; Yi et al., 2014). OBPs are responsible for the connection between the external environment and ORNs in the odorant-molecule recognition process. OBPs also mediates the first stage of the physiological process involved in the sensing of the external environment, by insects (Willett and Harrison, 1999; Laughlin et al., 2008; Pelosi et al., 2014; Leal and Leal, 2015).

OBPs belong to a class of small water-soluble proteins that are impregnated in the sensillum lymph at extremely high concentrations (up to 10 mM; Vogt and Riddiford, 1981; Klein, 1987; Steinbrecht et al., 1992). The first insect OBP was identified in the antennae of male *Antheraea ployphemus*. By using a radiolabeled photo-affinity analog, this protein was designated as a pheromone binding protein (PBP) as it specifically bound to the female sex pheromone *E*6, *Z*11-hexadecadienyl acetate (Vogt and Riddiford, 1981). Since then, OBPs have been discovered in various insect orders (Hansson and Stensmyr, 2011; Antony et al., 2018; Fleischer et al., 2018). Lepidopteran OBPs are usually subdivided into three subfamilies including PBPs, general OBPs, and antennal binding proteins (ABPX), on the basis of amino-acid sequence homologies (Hekmat-Scafe et al., 2002). The PBPs are located in the sensilla trichodea and exhibit specific binding to female sex pheromones (Bette et al., 2002; Lautenschlager et al., 2007). GOBPs (further classified as GOBP1 and GOBP2) are primarily distributed in the sensilla basiconica and their function is mainly involved in the detection of general odorants (e.g., host plant volatiles; Vogt et al., 2002; Nardi et al., 2003; Maida et al., 2005; Liu et al., 2015). In some Lepidopteran species, GOBP2 also showed high-binding affinities to sex pheromones in addition to general odorants (Liu et al., 2010, 2012; Li et al., 2016a). ABPX are more divergent among insects and its functions may play a similar role than PBPs or GBOPs in the discrimination and transportation of semiochemicals (Tian et al., 2018).

The binding affinities of insect OBPs to odorant molecules have been measured via fluorescence competitive binding assays with N-phenyl-1-naphthylamine (1-NPN) as a probe (Pelosi et al., 2006; Zhou, 2010). For example, *Helicoverpa armigera* HarmOBP17 and HarmOBP18 have strong binding capacities to β-ionone (Li et al., 2013). *Locusta migratoria* LmigOBP1 exhibited specific-binding affinities to pentadecanol and 2-pentadecanone, where Asn74 formed the key binding site in these two ligands (Jiang et al., 2009). *Grapholita molesta* GmolGOBP2 had specific binding ability to the minor sex pheromone component 1-dodecanol (Li et al., 2016a). The fluorescence competitive binding assay is only *in vitro* and the binding functions of OBPs still need to be verified further by experiments *in vivo*. RNAi experiments demonstrated that OBPs are indispensable in the olfactory communication of insects. For example, the electroantennogram (EAG) responses of female *Adelpocoris lineolatus* to tridecanal and 1-hexanol were drastically reduced after the double-stranded RNA (dsRNA) of *Alin*OBP4 was injected into both female and male adult insects (Zhang et al., 2017). The EAG values of AgosOBP2-dsRNA-treated *Aphis gossypii* to cotton-derived volatiles were remarkably lower than those of non-injected controls (Rebijith et al., 2016). By silencing the RferOBP1768 gene of *Rhynchophorus ferrugineus*, the adults apparently lose the ability to recognize the aggregation pheromone compounds 4-methyl-5-nonanol and 4-methyl-5-nonanone (Antony et al., 2018).

The oriental fruit moth *Grapholita molesta*, is a destructive fruit pest species that causes considerable economic losses in fruit yields on a global scale (Rothschild and Vickers, 1991). The first three moth generations mainly infest peach shoots in the early growing season, whereas the third generation begins to shift and attack pear and apple orchards in the late growing season. The migration of the adults is predominantly guided by the change in volatile components emitted by these host plants (Myers et al., 2007). At present, monitoring *the G. molesta* mainly depends on the pheromone trapping of male moths. However, the females have multiple mating abilities and their flight capabilities are three to six times greater than that of males and the females also have higher mating rates in the pheromone trapping orchards (Hughes and Dorn, 2002; Il'ichev et al., 2007; Zhang et al., 2012). Therefore, a strategy to monitor both female and male moths, based on olfactory cues emitted from host plants, is desirable. For example, a three-compound mixture of (Z)-3-hexen-1-ol, (Z)-3-hexen-1-yl acetate, and benzaldehyde in proportion 1:4:1 can attract female *G.molesta* just as well as the natural blend from peach shoots can (Natale et al., 2003).

In this study, *GmolOBP*7 was cloned using specific-primers based on the annotated unigene from the antennal transcriptome of *G. molesta*. qRT-PCR was performed to determine the expression patterns of *GmolOBP*7 in different tissues, genders, and developmental stages of the *G. molesta*. The binding affinities of the rGmolOBP7 with sex pheromone components and the host plants' volatiles, were measured via fluorescence binding assays. Furthermore, the ligand-binding functions of GmolOBP7 were further verified *in vivo* by knocking down the *GmolOBP*7 gene. The olfactory mechanism of the oriental fruit moth was further explicated to provide a theoretical basis for the design and implementation of control strategies against this fruit pest.

## Materials and Methods

### Insect Samples

*G. molesta* individuals were obtained from the College of Plant Protection, Northwest A&F University, Yangling, Shaanxi, China. The laboratory colony has been maintained for more than 90 generations. The larvae were reared on an artificial diet at 25 ± 1°C, 70% ± 5% RH under a day/night cycle of 15:9, until pupation (Du et al., 2010). After pupation, male, and female pupae were placed in separate glass tubes and maintained under the same conditions described above. The adults were fed 5% honey solution daily. To detect the tissue distribution of *GmolOBP*7 in adult moths, various tissues (including antennae, heads without antennae, thoraces, abdomens, legs, and wings) were collected from 3-day-old males and females and immediately transferred to 1.5 mL Eppendorf tubes immersed in liquid nitrogen. All samples were stored at −80°C prior to use. In order to determine the transcript level of GmolOBP7 in different developmental stages of the *G. molesta*, samples of eggs, larvae (including 1st, 2nd, 3rd, 4th, and 5th instars), pupae (including prepupae and later-pupae) and adults (including 1-d-old, 3-d-old, and 5-d-old adults) were collected and stored at −80°C prior to use.

### RNA Extraction, OBP Cloning, and Sequencing

Total RNA of all samples was extracted using a RNAiso Plus reagent (TaKaRa, Daian, China) according to the manufactures' instructions. The residual genomic DNA in the total RNA was removed using DNase I (Thermo Scientific, USA), and the first-strand cDNA was synthesized in accordance with the recommended protocols of the RevertAid First Strand cDNA Synthesis Kit (Thermo Scientific, USA). The products were stored at −80°C.

The predicted coding region of *GmolOBP*7 was cloned using specific-primers based on the annotated unigene from the antennal transcriptome of *G. molesta* (Table 1). The predicted results showed that GmolOBP7 had no signal peptide at the N-terminus of the amino acid sequence. In order to confirm whether we acquired the complete coding sequence of GmolOBP7, gene-specific primers were synthesized and used for 5' RACE (rapid amplification of cDNA ends; Table 1) Refers to a procedure in a previous study (Luo et al., 2011). A first 41-cycle touchdown PCR was performed using 5' RACE outer primers (named outer5F and outer5R; Table 1). A 25 μL PCR reaction system contained 12.5 μL of 2 × Super Pfx MasterMix (CWBIO, Beijing, China), 0.8 μL of each primer (10 μM), 1 μL of sample cDNA, and 9.9 μL of nuclease free water. The thermocycling program included denaturation at 95°C for 5 min, followed by 16 cycles of 30 s at 95°C, 1 min at 65 °C, and 2 min at 72 °C, and the annealing temperature was decreased 1°C each four cycles. The remaining 25 cycles consisted of 30 s at 95°C, 1 min at 61 °C, and 2 min at 72 °C, and a final extension step of 72°C for 10 min. The PCR products were diluted 80 times with sterilized ddH_2_O. The second 41-cycle touchdown PCR was conducted using 5' RACE inner primers (named inner5F and inter5R; Table 1) and the template with diluted PCR products. The reaction system and procedure was the same as the first round of PCR. The amplified product was purified with an Universal DAN Purification Kit (TianGen, Beijing, China), and cloned into the pMD®19-T cloning vector (TaKaRa, Dalian, China) and then transformed into DH5α *Escherichia coli* competent cells (TianGen, Beijing, China). Five positive clones were randomly selected for sequencing at the Aoke Biotech Company (Aoke, Xi'an, China).

**Table 1:** List of primers used in the current research.

**Primer name**	**Sequence (5^**′**^−3^**′**^)**	**PCR product size (bp)**
**For predicted ORFs**		
OBP7-forward	CCTTAAATGCCAAGAACAACT	525
OBP7-reverse	GCCTTTACAGGTCGAAACCAA	
**For 5′ RACE**		
Outer5F	AAGCAGTGGTATCAACGCAGAGTACGCGGGGGGGGGG	–
Outer5R	CAGCCATATCAGCTTTGGATGTTG	
inner5F	AAGCAGTGGTATCAACGCAGAGT	–
inner5R	GTACGACACCTTTAGTTGTTCTTG	
**For qRT-PCR**		
Actin-forward	CTTTCACCACCACCGCTG	156
Actin-reverse	CGCAAGATTCCATACCCA	
EF-1α-forward	AGGAGATCGAGCAACAGGAA	244
EF-1α-reverse	CACGACTCTCGGGACTTCTC	
OBP7-forward	AAGGTGTCGTACGCTGTCGT	154
OBP7-reverse	CACTTCATTCCGATTTCGTG	
**For prokaryotic expression**		
OBP7-forward	CGGGATCCACAACTAAAGGTGTCGTACGCT (BamHI)	504
OBP7-reverse	CCAAGCTTGGTTACAGGTCGAAACCAAACT (HindIII)	
**For synthesize dsRNA**		
OBP7i-forward	TAATACGACTCACTATAGGGAAAGACAACCCCATCACTGC	317
OBP7i-reverse	TAATACGACTCACTATAGGGACCAAACTGAGCAGCGTTTT	
GFP-forward	TAATACGACTCACTATAGGGGTGTTCAATGCTTTTCCCGT	315
GFP-reverse	TAATACGACTCACTATAGGGCAATGTTGTGGCGAATTTTG	

*The restriction endonucleases are in parentheses after each primer, and the restriction sites are underlined. “–” means the amplified DNA fragment is an unknown in size*.

### Sequence and Phylogenetic Analyses

The online programs of ORF Finder (http://www.ncbi.nlm.nih.gov/gorf/gorf.html), SignalP 4.0 (http://www.cbs.dtu.dk/services/SignalP/), and ExPASy server (https://web.expasy.org/compute_pi/) were used to predict the ORFs, signal peptides and the molecular weight and isoelectric point of mature protein of GmolOBP7, respectively. The amino-acid sequences were aligned using ClustalX 1.83 software. A phylogenetic tree was established by the MEGA6.0 software using the neighbor-joining method (NJ) with 1,000 bootstrap replications, and the tree was drawn using Adobe Photoshop CS5.

### Expression Analysis Using qRT-PCR

The expression levels of *GmolOBP*7, in different tissues and developmental stages of *G. molesta*, were measured via qRT-PCR. All qRT-PCR experiments were performed according to the MIQE Guidelines (Bustin et al., 2009). Specific primers were designed using the program Primer3-blast (https://www.ncbi.nlm.nih.gov/tools/primer-blast/), available online (Table 1). The elongation factor 1-alpha gene (*EF1*-α) (GenBank No: KT363835.1) and the β-actin gene (GenBank No: KF022227.1) were used as reference genes. The reactions were performed on a CFX96 Real-Time PCR Detection System (Bio-Rad, USA). Each amplification reaction was conducted using a 20 μL reaction system containing 10 μL of 2 × SYBR® Premix Ex Taq™ II mixture (TaKaRa, Dalian, China), 0.8 μL of each primer (10 μM), 1 μL of sample cDNA, and 7.4 μL of nuclease-free H_2_O. Samples without a template cDNA served as negative controls. To check reproducibility, test samples and negative controls were performed in triplicates. qRT-PCR was performed via initial denaturation at 95°C for 30 s, followed by 40 cycles of 95°C for 5 s, 60°C for 30 s and 72 °C for 30 s. The melting curves were used to examine primer specificity, and the standard curves were used to determinate the amplification efficiencies of target and reference genes. The expression levels of GmolOBP7 in different adult tissues and development stages were performed based on previous methods (Livak and Schmittgen, 2001; Liu et al., 2016). The expression level of all samples of the GmolOBP7 was calculated using the value of the amplification efficiency (*E*) and the value of the cycle threshold (*Ct*) (Equation 1). The normalized expression level of the tested samples was calculated by the geometric means of the expression level of the reference genes (β*-actin and EF-1*α) (Equation 2) (Vandesompele et al., 2002). The significant differences in different tissues and developmental stages were analyzed by the Tukey's HSD tests with a critical level of α = 0.05. The paired *t-test* was used to measure the impacts of the expression of *GmolOBP*7 between male and female moths. All the data were analyzed using SPSS 18.0 software (SPSS Inc., Chicago, IL, USA).

(1) $$\begin{array}{l} \text{Expression level}={\left(1+\text{E}\right)}^{-\text{Ct}} \end{array}$$

(2) $$\begin{array}{l} \text{Normalized expression level of target gene}\\ =\frac{{\left(1+E_{target}\right)}^{-Ct_{target}}}{\sqrt{{\left(1+E_{actin}\right)}^{-Ct_{actin}}\times {\left(1+E_{EF-1\alpha }\right)}^{-Ct_{EF-1\alpha }}}} \end{array}$$

### Expression Vector Construction

Specific primers with restriction enzyme sites were designed to clone the coding region of the GmolOBP7 (Table 1), and the PCR product was then cloned into the pMD®19-T cloning vector (TaKaRa, Dalian, China) and sequenced. The recombinant plasmid pMD®19-T/GmolOBP7 and the expression vector pET32a(+) (Novagen, Madison, WI, USA) were digested with the same restriction endonucleases, and the released DNA fragment was cloned into pET-32a(+) and then transformed into BL21 *E. coli* competent cells (Tiangen, Beijing, China). A positive clone containing pET32a(+)/GmolOBP7 was further confirmed by sequencing.

### Protein Expression and Purification

The overnight bacterial solution was diluted with 750 mL of LB medium (with 100 mg/mL ampicillin) and cultured at 37°C until its cell density reached a value of OD_60_ = 0.6. The cultures were induced by adding isopropyl-β-D-thiogalactoside (IPTG) at a final concentration of 0.5 mM for an additional 5 h at 37°C, 220 rpm. The bacterial cells were harvested by centrifugation (10 min at 8,000 rpm, 4°C), and the pellets were then sonicated in a lysis buffer (1 mM phenylmethanesulfonyl fluoride, 250 mM NaCl, and 20 mM Tris-HCl pH 7.4) and centrifuged again (13,000 g, 30 min, 4°C). A sodium dodecyl sulfate-polyacrylamide gel electrophoresis (SDS-PAGE) analysis revealed that the rGmolOBP7 was mainly present in supernatants. The supernatant of rGmolOBP7 was enriched by a Ni-NTA His·Bind Resin column (7 sea Pharmatech Co., Shanghai, China) in accordance with the manufacturer's instructions. To avoid the effects of His-tag on subsequent experiments, the His-tag was cleaved by a recombinant enterokinase (NEB, Beijing, China) and removed using the column mentioned above. The target protein was purified using affinity chromatography, and the concentration was determined by the BCA protein kit (Beyotine, Shanghai, China).

### Fluorescence Binding Assays

Fluorescence intensity was detected on a spectrophotofluorometer (F-4500, Hitachi, Japan) at room temperature using a quartz cuvette with a 1 cm light path. The silt width of excitation and emissions were all 10 nm. The fluorescence probe 1-NPN was excited at 337 nm and the emission spectra were recorded between 370 and 550 nm. Four sex pheromone components and 31 volatiles, derived from peach shoots and pear fruits, were selected for binding assays (Table 2). The probe 1-NPN and tested ligands were all dissolved in spectrophotometric-grade methanol to obtain a 1 mM stock solution. The binding affinity of rGmolOBP7 with 1-NPN was measured by adding aliquots of 1-NPN to a 2 μM protein solution (diluted with 20 mM Tris-HCl pH 7.4) to final concentrations of 0 to 18 μM.

**Table 2:** Binding affinities of GmolOBP7 to various ligands were measured via competitive binding assays using 1-NPN as a fluorescent probe.

**Chemical compounds**	**Molecular weight**	**Formula**	**Purity(%)**	**IC_**50**_ (μM)**	**K_**i**_ (μM)**
**SEX PHEROMONES**					
(*Z*)-8-dodecenyl acetate	226.36	C_14_H_26_O_2_	>95.0(AR)	>35	>35
(*E*)-8-dodecenyl acetate	226.36	C_14_H_26_O_2_	>95.0(AR)	>35	>35
(*Z*)-8-dodecenyl alcohol	200.34	C_12_H_24_O	>98.0(AR)	>35	>35
1-Dodecanol	186.34	C_12_H_26_O	>99.0(GC)	10.73 ± 0.85	7.48
**ALDEHYDES**					
(*E*)-2-Hexenal	98.15	C_6_H_10_O	98.0(AR)	24.38 ± 1.53	16.99
Hexanal	100.16	C_6_H_12_O	>95.0(AR)	27.40 ± 1.59	19.10
Benzaldehyde	106.12	C_7_H_6_O	≥99.5(GC)	20.19 ± 0.27	14.07
Heptanal	114.18	C_7_H_14_O	97.0(AR)	15.66 ± 0.64	10.92
Nonanal	142.24	C_9_H18O	95.0(AR)	28.56 ± 3.38	19.91
Decanal	156.26	C_10_H_20_O	97.0(AR)	18.16 ± 1.27	12.66
Lauraldehyde	184.32	C_12_H_24_O	98.0(AR)	6.18 ± 0.43	4.31
Tetradecanal	212.37	C_14_H_28_O	95.0(AR)	>35	>35
**ALCOHOLS**					
(*Z*)-3-Hexen-1-ol	100.16	C_6_H_12_O	98.0(AR)	17.62 ± 0.52	12.28
1-Hexanol	102.18	C_6_H_14_O	>98.0(AR)	24.90 ± 1.28	17.35
Benzyl alcohol	108.13	C_7_H_8_O	>99.0(GC)	>35	>35
Linalool	154.25	C_10_H_18_O	>99.0(GC)	21.73 ± 1.11	15.15
1-Decanol	158.29	C_10_H_22_0	>99.0(GC)	>35	>35
1-Tetradecanol	214.39	C_14_H_30_O	>98.0(AR)	>35	>35
Nerolidol	222.37	C_15_H_26_O	>98.0(AR)	>35	>35
1-Hexadecanol	242.44	C_16_H3_4_O	>99.0(GC)	>35	>35
**ESTERS**					
Butyl acetate	116.16	C_6_H_12_O_2_	99.0(AR)	18.10 ± 1.57	12.62
Pear ester	128.17	C_7_H_12_O_2_	≥97.0(GC)	3.62 ± 0.25	2.52
(*Z*)-3-Hexenyl acetate	142.2	C_8_H_14_O_2_	>97.0(GC)	19.64 ± 0.27	13.69
Butyl butyrate	144.22	C_8_H_16_O_2_	>99.0(GC)	16.27 ± 01.71	11.34
Methyl salicylate	152.15	C_8_H_8_O_3_	≥99.0(GC)	14.08 ± 0.22	9.81
Butyl hexanoate	172.27	C_10_H_20_O_2_	≥99.5(GC)	24.40 ± 0.77	17.01
Methyl jasmonate	224.32	C_13_H_20_O_3_	98.0(AR)	18.73 ± 0.22	13.05
Methyl myristate	242.41	C_15_H_30_O_2_	≥95.0(GC)	>35	>35
Methyl palmitate	270.45	C_17_H_34_O_2_	≥98.0(GC)	>35	>35
**TERPENES**					
α-Pinene	136.23	C_10_H_16_	98.0(AR)	17.09 ± 1.20	11.91
α-Ocimene	136.23	C_10_H_16_	≥90.0(AR)	11.12 ± 0.20	7.75
(-)-Camphene	136.23	C_10_H_16_	≥98.0(GC)	>35	>35
β-Caryophyllene	204.36	C_15_H_24_	97.0(AR)	>35	>35
**BENZONITRILES**					
Benzonitrile	103.12	C_7_H_5_N	>99.0(AR)	17.93 ± 1.29	12.50
Lemonile	149.23	C_10_H_15_N	98.0(AR)	>35	>35

*More than >35 μM indicates that the IC_50_ and K_i_ values are above the concentration ranges tested*.

To test the binding affinities of GmolOBP7 to various ligands, 2 μM solutions of rGmolOBP7 and 1-NPN were titrated with the 1 mM solution of each ligand to a final concentration of 0–14 μM for sex pheromones and 0–35 μM for host volatiles. The corresponding florescence intensity values were collected as three independent measurements. The binding constant (K_1−NPN_) of 1-NPN to rGmolOBP7 was calculated using GraphPad Prism 5 software (GraphPad Software, Inc.) via nonlinear regression for a unique site of binding. The dissociation constant (*K*_*i*_) of each ligands competitive binding to rGmolOBP7, were calculated from the corresponding IC_50_, by using the equation K*i* = [IC_50_]/(1+[1-NPN]/K_1−NPN_), where [1-NPN] is the free concentration of 1-NPN, and K_1−*NPN*_ is the dissociation constant of the complex protein/1-NPN.

### dsGmolOBP7 and dsGFP Synthesis

The specific-primers, including T7 RNA polymerase promoter, were designed to clone DNA fragments of GmolOBP7 for 317 bp and green fluorescent protein (GFP) for 315 bp (Table 1). The purified PCR products were used as a template for dsRNA (dsGmolOBP7 and dsGFP) synthesis using the T7 RiboMAXTM Express RNAi System kit (Promega, USA) according to the manufacturer's instructions. The purified dsRNA was quantified via spectrophotometry (SimpliNano, GE, USA), and the dsRNA integrity was monitored by electrophoresis on 1.5% agarose gel. Each dsRNA sample was dissolved in nuclease-free water to a final concentration of 3,500 ng/μL.

### dsRNA Microinjection

Based on the expression patterns observed at different insect stages, 5-day-old *G. molesta* pupae (later-pupae) were selected to receive a dsRNA microinjection. The conjunctivum between the prothorax and mesothorax, the conjunctivum between the mesothorax and metathorax and the conjunctivum between the thorax and abdomen were initially selected as putative injection sites. Then, 39, 69, and 138 nL of RNase-free H_2_O were injected into different conjunctiva, respectively. A total of 69 nL (approximately 241.5 ng dsRNA) of dsGmolOBP7 or dsGFP was injected into the appropriate injection sites of each 5-day-old pupa, by using a PL1-100 Pico-Injector (Harvard Apparatus, Holliston, MA, USA) operated by an MP-255 Micromanipulator (Sutter, Novato, CA, USA). Each type of dsRNA was injected into 600 male and 600 female moths. The heads (with antenna) of 1-, 2-, 3-, and 4 day-old adults were dissected and immediately stored at −80°C prior to use. The total RNA and first-strand cDNA were obtained in accordance with previous methods. The specimens were used for qRT-PCR analysis to test the reduction in *GmolOBP*7 transcription. Experiments were performed with three biological replicates and three technical replicates.

### EAG Assays

EAG responses of dsRNA-injected (including dsGmolOBP7 and dsGFP) moths and non-injected controls, to sex pheromones and host plant volatiles were detected using Electroantennography. All stimulants were diluted with liquid paraffin to the final concentration of 10 mg/mL. Liquid paraffin and *cis*-3-hexenyl acetate were used as the blank and reference control, respectively. Both ends of adult antennae were cut and blocked with a drop of Spectra® 360 Electrode Gel (Parker Laboratories, Fairfield, USA). The basal section was connected to the reference electrode while the distal end was linked to the recording electrode. A filtered humidified air stream was delivered by a Syntech stimulus controller (CS55 model, Syntech, Germany) at a constant flow rate of 50 cm/s, and the time of stimuli flow was 0.5 s. Filter paper strips (0.6 cm × 4.5 cm) were dripped with 15 μL of chemical solution as a stimulus source and inserted into a 1.5 mL micropipet tip. A set of stimulants consisted of four sex pheromones and five host-plant volatiles which can be strongly bound with rGmolOBP7. Each antenna measured the group of randomly arranged stimulants described above. The antennae were stimulated one time with liquid paraffin and *cis*-3-hexenyl acetate and dissolved in solvent, before and after each group stimulation, in order to ensure the tested antenna were activated and the connecting pipe was not contaminated by stimulants. Recordings per stimulant were taken and the antennal responses were recorded. Eight male and female antennae were measured for each stimulant. The paired *t-test* was used to determine whether the difference in EAG values were significant between the dsRNA injected moths (dsGmolOBP7 and dsGFP) and the non-injected control. All the data were analyzed using SPSS 18.0 software (SPSS Inc., Chicago, IL, USA).

## Results

### Identification of *GmolOBP*7 in *G. molesta*

**Figure 1:**
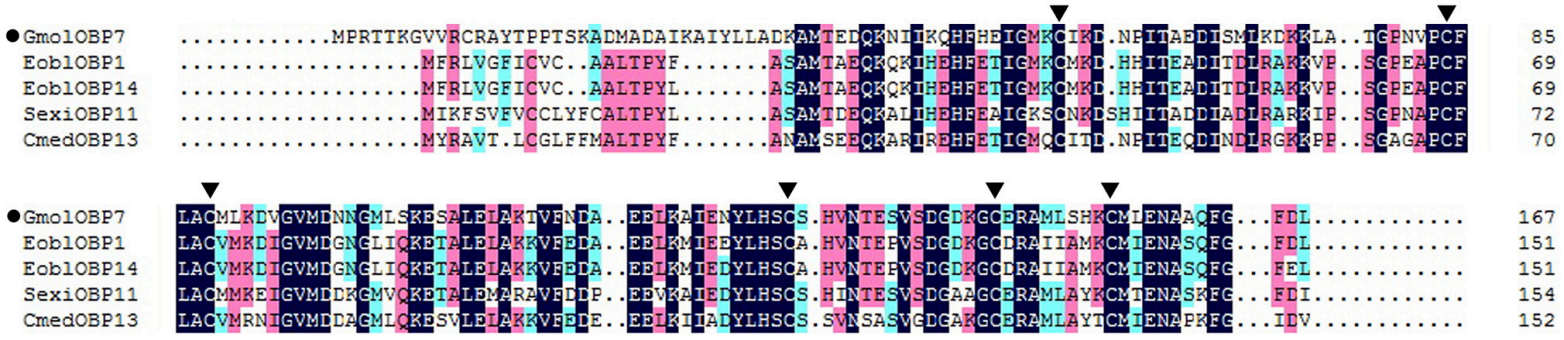
Sequence alignment of GmolOBP7 and other OBPs from Lepidopteran insects. The names and GeneBank accession numbers of five OBPs are as follows: *Graphita molesta* (GmolOBP7, MF066359); *Ectropis obliqua* (EoblOBP1, ANA75015.1; EoblOBP14, ALS03862.1); *Spodoptera exigua* (SexiOBP11, AGP03457.1); and *Cnaphalocrocis medinalis* (CmedOBP13, ALT31643.1). The six conserved cysteines were marked by a triangle with a black background.

**Figure 2:**
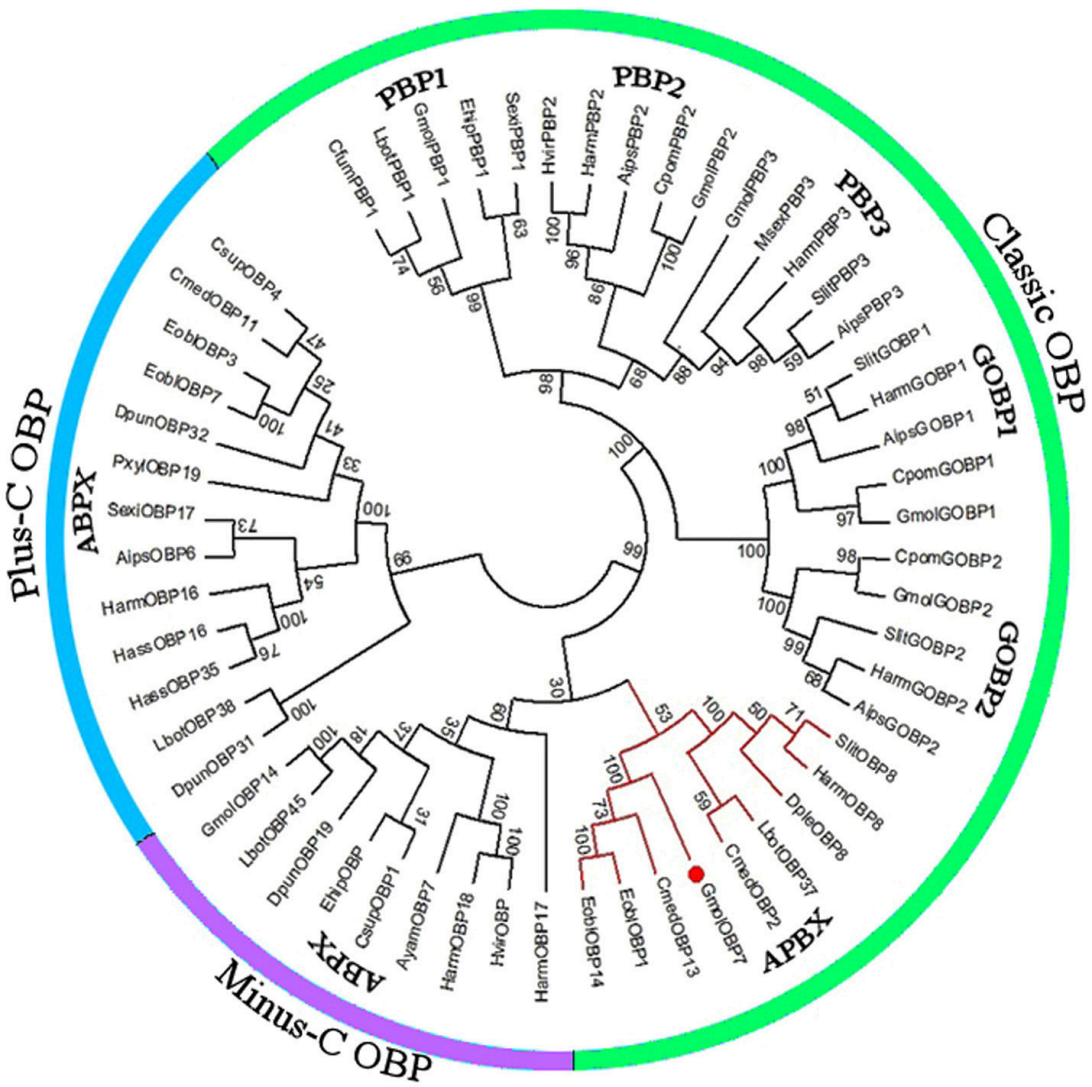
Phylogenetic tree of OBPs from *Grapholita molesta* and other Lepidoptera via the neighbor-joining method with a bootstrap replication of 1,000. The unrooted tree was constructed using MEGA 6.0, based on the sequence alignment produced using ClustalX 1.83 software. The species and GenBank accession numbers of the sequence are as follows: A*grotis ipsilon* (AipsGOBP1, AFM36759.1; AipsGOBP2, AFM36760.1; AipsPBP2, JQ822241; Aips PBP3, JQ822242; AipsOBP6, AGR39569.1); *Antheraea yamamai* (AyamOBP7, ADO95155.1); *Chilo suppressalis* (CsupOBP1, AGK24577.1; CsupOBP4, AGK24580.1); *Choristoneura fumiferana* (CfumPBP1, AAF06127.1); *Cnaphalocrocis medinalis* (CmedOBP2, AFG73000.1; CmedOBP11, AFG72998.1; CmedOBP13, ALT31643.1); *Danaus plexippus* (DpleOBP8, OWR42851.1); *Dendrolimus punctatus* (DpunOBP19, ARO70178.1; DpunOBP31, ARO70190.1; DpunOBP32, ARO70191.1); *Ectropis obliqua* (EoblOBP1, ANA75015.1; EoblOBP3, ANA75017.1; EoblOBP7, ALS03855.1; EoblOBP14, ALS03862.1); *Eogystia hippophaecolus* (EhipPBP1, AOG12881.1; EhipOBP, AOG12871.1); *Graphita molesta* (GmolGOBP1, JN857939; GmolGOBP2, JN857940; GmolPBP1, MF066363; GmolPBP2, KF365878; GmolPBP3, KF365879; GmolOBP7, MF066359; GmolOBP14. MF066361); *Helicoverpa armigera* (HarmGOBP1, AAL09821.1; HarmGOBP2, CAC08211.1; HarmPBP2, AEB54583.1; HarmPBP3, AAO16091.1; HarmOBP8, AEB54589.1; HarmOBP16, AFI57165.1; HarmOBP17, AFI57166.1;HarmOBP18, AFI57167.1); *Helicoverpa assulta* (HassOBP16, AGC92791.1; HassOBP35, ASA40073.1); *Heliothis virescens* (HvirPBP2, CAL48346.1; HvirOBP, ACX53795.1); *Lobesia botrana* (LbotPBP1, AXF48748.1; LbotOBP37, AXF48734.1; LbotOBP38, AXF48735.1; LbotOBP45, AXF48742.1); *Manduca sexta* (MsexPBP3, AAF16703.1); *Plutella xylostella* (PxylOBP19, ANC60176.1); *Spodoptera litura* (SlitPBP, ABQ84981.1; SlitGOBP2, AKI87961.1; SlitPBP3, GU082321; SlitOBP8, AKI87969.1); *Cydia pomonella* (CpomGOBP1, AFP66957.1; CpomGOBP2, AFP66958.1; CpomPBP2, AFL91693.1) and *Synanthedon exitiosa* (SexiPBP1, AAF06142; SexiOBP17, AKT26495.1).

We obtained the ORF of GmolOBP7 (GeneBank No. MF066359) using ordinary PCR and 5' RACE PCR based on the annotated unigene from the antennal transcriptome of *G. molesta*. The ORF of *GmolOBP*7 is 504 bp in length and encodes 167 amino acids (Figure S1). *GmolOBP*7 possesses a common characteristic of known classical-OBPs with six-conserved cysteine motifs (Figure 1). The mature protein has a predicted molecular weight of 21.47 kDa and a theoretical pI of 6.85. The SignalP 4.1 server prediction indicated that *GmolOBP*7 did not have signal peptides at the N-terminus of the amino acid sequence. GmolOBP7 shares the highest identities with *Ectropis obliqua* EoblOBP1 and *Spodoptera exigua* SexiOBP11, with an identity of 67 and 64%, respectively. Phylogenetic analysis showed that *GmolOBP7* were clustered into a small branch close to ABPXs from *Ectropis obliqua* and *Cnaphalocrocis medinalis* (Figure 2).

### Expression Profiles of *GmolOBP*7 in *G. molesta*

**Figure 3:**
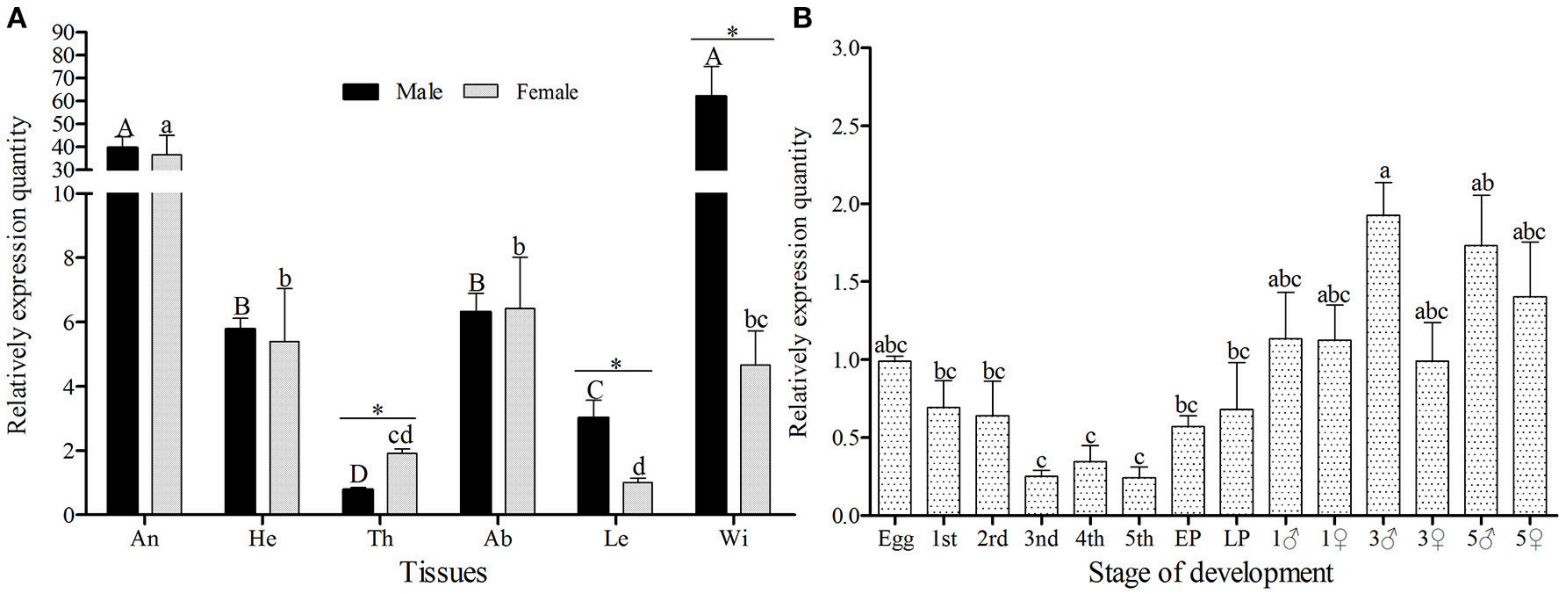
Expression profiles of *GmolOBP*7 in different tissues **(A)** and developmental stages **(B)** of male and female moths. An, antennae; He, heads; Th, thoraces; Ab, abdomens; Le, Legs; Wi, Wings; 1st, first-instar larvae; 2nd, second-instar larvae; 3rd, third-instar larvae; 4th, fourth-instar larvae; 5th, fifth-instar larvae; Pup, prepupae; Later Pup, 5-d-old pupae; 1♂, 1-d-old adult males; 1♀, 1-d-old adult females; 3♂,3-d-old adult males; 3♀, 3-d-old adult females; 5♂, 5-d-old adult males; 5♀, 5-d-old adult females. Different lowercase and capital letters indicate significantly different expression levels among different tissues of female and male, respectively (Tukey's test, α = 0.05). Asterisks indicate significant different expression levels of GmolOBP7 between two sexes in the same tissue (Independent *t*-test, α = 0.05).

The amplification efficiency and melting curve of target and reference genes showed that the two specific-primers were similar in amplification speed and no nonspecific products were produced (Figure S2), therefore, the primers can be used for relative quantification. *GmolOBP*7 was detected in all tested tissues of both female and male moths (Figure 3A), but the expression quantity was higher in the wings of males and antennae of both sexes, than that in other tested tissues. The expressed quantity of GmolOBP7 was different between males and females, with the quantity in male wings and legs being significantly higher than in female wings and legs (with about 13.31 and 2.99 times differences, respectively), and was higher in female thoraces than in male thoraces (about 2.43-fold higher). The expression levels of GmolOBP7 in different developmental stages were performed by qRT-PCR (Figure 3B), the highest expression quantity was found in adults, followed by the eggs, 1st instar larvae, and later pupae (5-day-old pupae), the expression levels of *GmolOBP*7 were extremely low in second- to fourth-instar larvae.

### Expression and Purification of *GmolOBP*7

**Figure 4:**
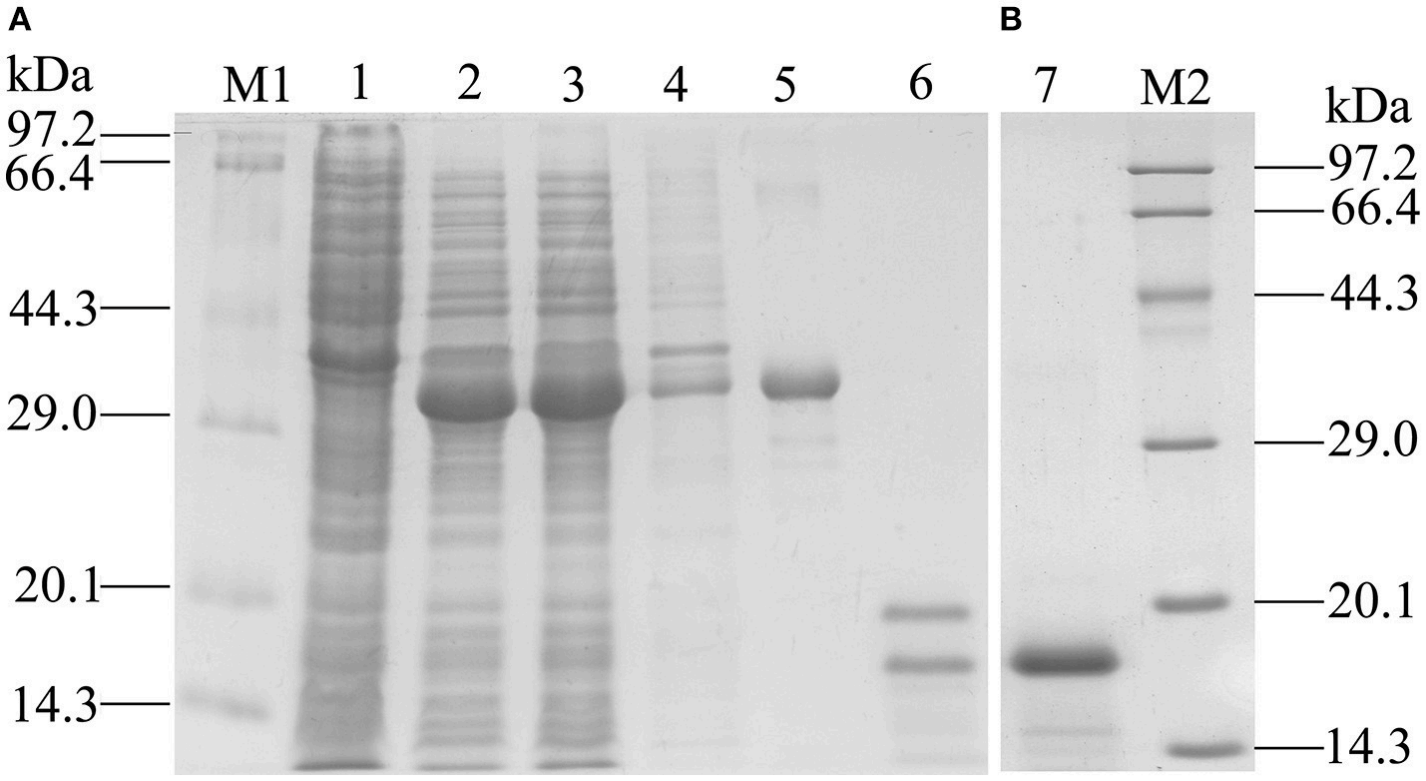
SDS-PAGE analysis of recombinant GmolOBP7. **(A)** Expression and purification of GmolOBP7; **(B)** Purified GmolOBP7 protein after removed the His-tags. M1 and M2: standard protein maker; 1. Noninduced pET32a(+)/GmolOBP7; 2. Induced pET32a(+)/GmolOBP7; 3. Supernatant-induced pET32a(+)/GmolOBP7; 4. Precipitate of induced pET32a(+)/GmolOBP7; 5. Purified protein of pET32a(+)/GmolOBP7; 6. Digestion products of the purified pET32a(+)/GmolOBP7 using enterokinase; 7. Re-purification of GmolOBP7 after the removal of His-tags.

Recombinant GmolOBP7 (rGmolOBP7) was successfully expressed in *E. coli* as a soluble protein (Figure 4A). After being purified, about 38 kDa recombinant protein with His-tag was obtained. To avoid the effect by the His-tag on subsequent binding assays, the His-tag of pET32(a+)/GmolOBP7 was cleaved by enterokinase and then removed by Ni-NTA His·Bind Resin and SDS-PAGE analysis showed that rGmolOBP7 had a higher purity after being purified for a second time via affinity chromatography (Figure 4B). The purified recombinant protein was then tested for the binding affinity with various ligands.

### Fluorescent Binding Assay of GmolOBP7

**Figure 5:**
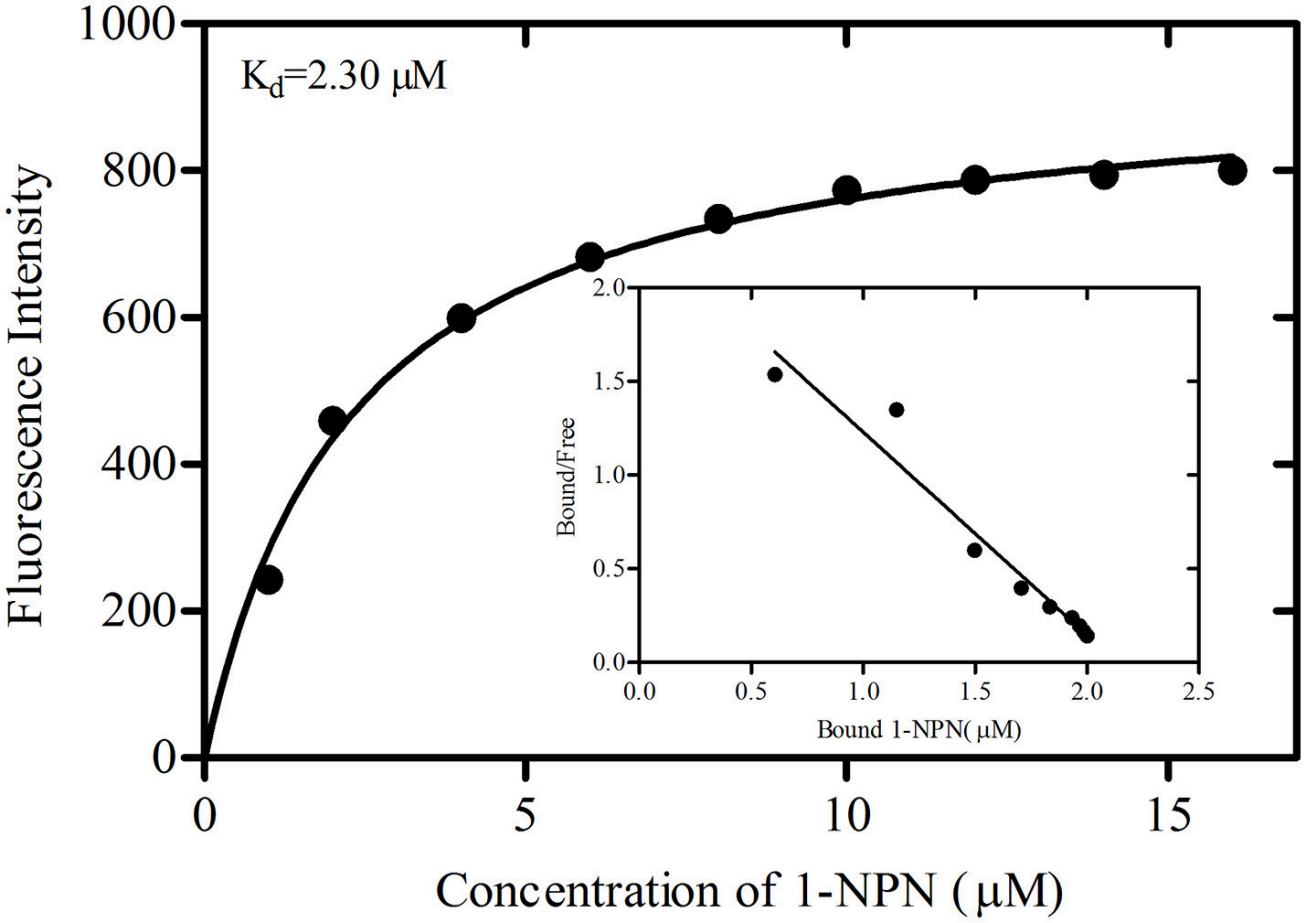
Binding curve of 1-NPN and Scatchard plots for recombinant GmolOBP7. A 2 μM solution of protein in 20 mM Tris-HCl buffer (pH 7.4) was titrated with 1 mM 1-NPN solution to final concentrations of 1 to 18 μM, and the emission spectra were recorded between 370 and 550 nm. The dissociation constant (K_d_) of GmolOBP7 was 2.30 μM.

The binding curve and the derived Scatchard plot showed that the dissociation constant for rGmolOBP7 with the fluorescence probe 1-NPN was 2.30 μM (Figure 5). This result suggests the existence of a single binding site and the absence of an allosteric effect between the recombinant protein and the fluorescence probe.

**Figure 6:**
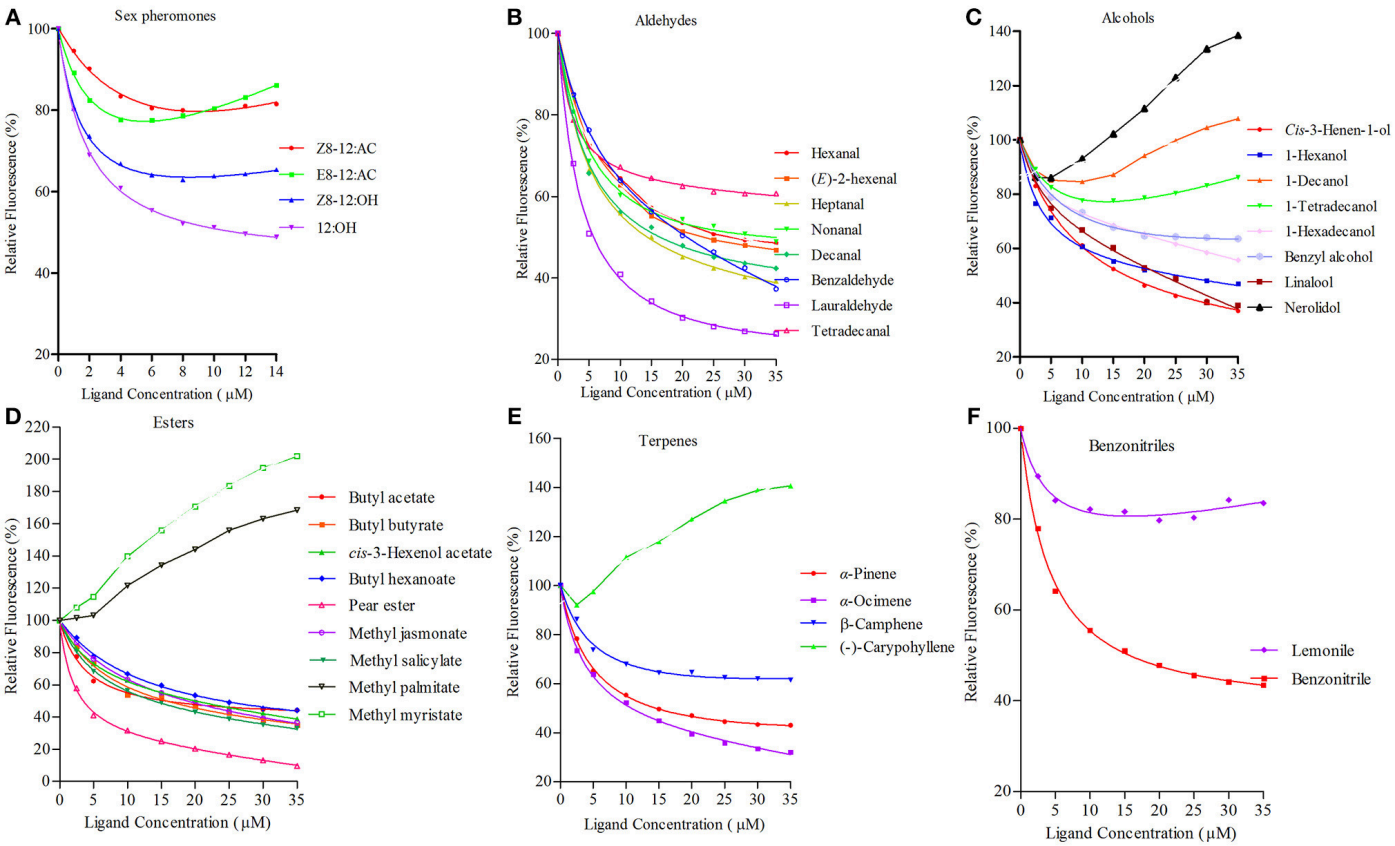
Binding curves of recombinant GmolOBP7 to a series of tested ligands. **(A)** sex pheromones; **(B)** aldehydes; **(C)** alcohols; **(D)** esters; **(E)** terpenes; **(F)** nitriles. The protein was diluted to a fixed concentration of 2 μM and then titrated with 1 mM of each competing ligand to a concentration of 0–14 μM for sex pheromones and 0–35 μM for host-plant volatiles. Fluorescence intensities are displayed as the percentage of the initial fluorescence. The calculated dissociation constants for all the ligands are listed in Table 2.

rGmolOBP7 showed broad binding properties with 35 putative ligands, 21 out of 35 ligands succeeded in displacing 1-NPN from the GmolOBP7/1-NPN complex by half, at concentrations up to 35 μM. The IC50 values and the calculated binding constants (Ki) are shown in Table 2. rGmolOBP7 exhibited high binding affinity to the minor sex pheromone component 1-dodecanol (12:OH) with a K_i_ value of 7.48 μM. However, rGmolOBP7 did not bind to the major sex pheromone components (Z)-8-dodecenyl acetate (*Z*8-12:Ac), (*E*)-8-dodecenyl acetate (*E*8-12:Ac) and (Z)-8-dodecenyl alcohol (*Z*8-12:OH) (Figure 6A). rGmolOBP7 had the strongest binding capacity to pear ester [Ethyl (*E, Z*)-2,4-decadienate] (with K_i_ value of 2.52 μM) in various volatiles emitted from peach shoots and pear fruits (Figure 6D). Additionally, rGmolOBP7 showed pronounced binding affinities with lauraldehyde and α-Ocimene with *K*_*i*_ values of 4.31 and 7.75 μM, respectively, (Figures 6B,E). rGmolOBP7 displayed intermediate binding affinities to some other aldehydes, alcohols, esters, and terpenes and nitriles with *K*_*i*_ values of 9.81 to 19.91 μM (Figure 6).

### Effect of RNAi Treatment on the Expression Level of *GmolOBP*7

Pilot experiments showed low eclosion rates when the pupae were injected with 138 nL of water at all putative conjunctiva (< 30%). The emergence rates of the pupae injected with 39 and 69 nL of water at different candidate conjunctiva ranged from 78.6 to 84.2%. Thus, the conjunctivum between the prothorax and mesothorax was selected as the appropriate injection site, and 69 nL was selected as the appropriate dosage.

**Figure 7:**
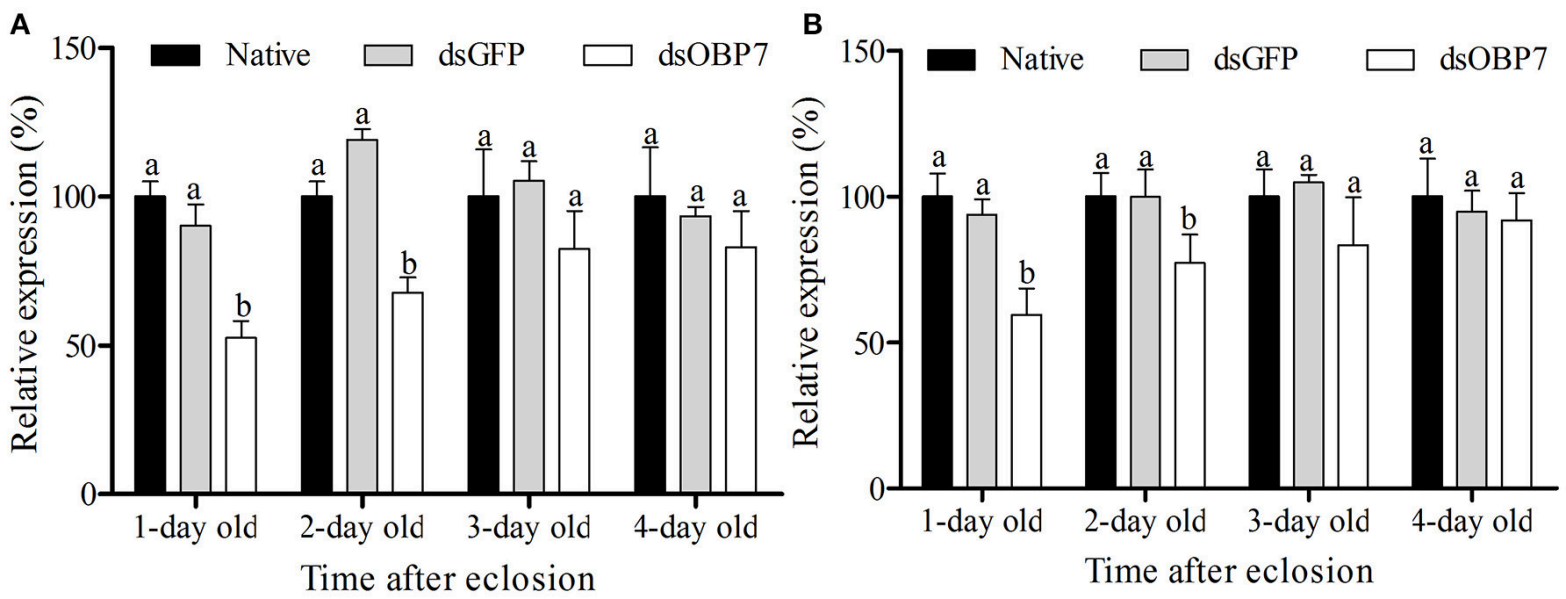
Expression level of GmolOBP7 in dsRNA-treated and no-injected moths at different ages of adult moths. **(A)** male moths; **(B)** female moths. About 240 ng (69 nL) of GmolOBP7 dsRNA and dsGFP were injected into 5-day-old pupae, respectively. Asterisk Different letters indicate significantly different expression levels of GmolOBP7 between dsRNA-treated moths and non-injected moths (independent *t*-test, α = 0.05).

qRT-PCR analysis revealed that the transcription levels of dsGFP-injected moths had no significant differences compared to the non-injected male and female moths. The transcript levels of *GmolOBP*7 decreased to 52.57% (with 1-d eclosion) and 67.68% (with 2-d eclosion) in GmolOBP7-dsRNA-injected males compared to that in the dsGFP-treated and non-treated controls (Figure 7A), and decreased to 59.50% (with 1-d eclosion) and 77.17% (with 2-d eclosion) in GmolOBP7-dsRNA-injected females compared to that in the controls (Figure 7B). The transcript levels of GmolOBP7 in dsRNA-treated moths were increased to normal values after 3-days of eclosion. Thus, 1-day-old adult moths were selected for subsequent EAG assays.

### Electrophysiological Experiments

**Figure 8:**
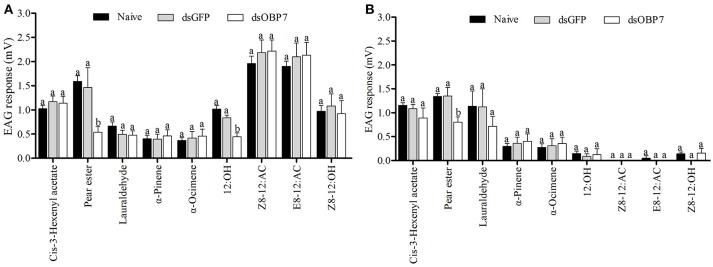
Electrophysiological response of *Grapholita molesta* to nine stimulants after RNAi knockdown. **(A)** male and **(B)** female moths. dsGmolOBP7 and dsGFP indicates treated moths injected with GmolOBP7 dsRNA and GFP dsRNA, respectively. Each treatment included 8 months, the antennae were stimulated with 15 μL volatile compound dissolved in liquid paraffin (ck), and ck and (*Z*)-3-hexenyl acetate were used to stimulate the antennae before and after a group of volatiles stimulation. Different letters indicate significant differences between dsRNA-treated moths and non-injected moths (independent *t*-test, α = 0.05).

The EAG response values of dsGFP-treated moths to nine tested stimulants had no significant differences to the non-injected male and female moths (Figure 8). The *t*-tests showed that the responses of both female and male moths, to pear ester were significantly reduced (*P* < 0.05) after injection with GmolOBP7-dsRNA, and the response value of male moths to 12:OH was also significantly decreased. However, the response to (Z)-8-dodecenyl acetate, (*E*)-8-dodecenyl acetate, (Z)-8-dodecenyl alcohol, (*Z*)-3-hexenyl acetate, lauraldehyde, α-pinene, and α-ocimene was not significantly different between dsRNA-treated moths and the non-injected control.

## Discussion

The OBP family genes are composed of many highly differentiated subfamily genes, the olfactory function of those OBPs highly-expressed in the antennae, such as GOBPs and PBPs, has been studied extensively (Zhou et al., 2009; Yin et al., 2012; Khuhro et al., 2017). However, the ligand-binding capacities of OBPs which had no antenna-specific expression or were lowly-expressed in antennae, remains poorly understood. We identified 26 OBPs from the antennal transcriptome of *G. molesta* (Li et al., 2015), the antenna-highly-expressed OBPs (PBP1-3, GOBP1-2, OBP8, OBP11, and OBP15) all with their preferred odorant ligands, such as GmolPBP2, which can bind specifically to the major sex pheromone components *Z*8-12:Ac and *E*8-12:Ac (Song et al., 2014), GmolGOBP1 have strong binding affinities to the major sex pheromone component Z8-12:OH and plant volatile decane (Li et al., 2016a), while hexanal was the preferred ligand of GmolOBP15 (Li et al., 2016b). We speculated that the relatively low-expression antennal OBPs may play a role in capturing and transporting the specific compounds of the host plant volatiles. The RPKM (reads per kilobase per million mapped reads) value of GmolOBP7 ranked 18th in 28 OBPs (Li et al., 2015), and can be expressed in soluble forms in a prokaryotic system. Therefore, we selected GmolOBP7 to evaluate its role in perceiving and recognizing the trace components emitted from peach shoots and pear fruits.

The expression profiles of olfactory-related genes in different tissues and sexes can provide clues to understand their physiological function (Ju et al., 2014). Numerous experiments have revealed that the antennae-enriched OBPs play an important role in detecting sex pheromones and host plant compounds (Sun et al., 2014; Yang et al., 2016; Khuhro et al., 2017). GmolOBP7 was expressed at relatively high levels in the antennae compared to other tissues, and might have potential functions in olfactory chemoreception. *G. molesta* reached the peak of mating after emergence (2- to 3-days) after which the flourishing period of oviposition of 3- to 5-day-old female adults occurred. The transcript levels of GmolOBP7 were slightly higher in 3-day-old male adults than in females of the same age, and the expressed levels were enhanced slightly in 3-day-old female adults. These expression characteristics implied the GmolOBP7 may be involved in the detection of sex pheromones and host-plant volatiles. In addition to antennae, GmolOBP7 was also abundantly expressed in the male wings of *G. molesta*, while similar expression profiles were found in BodoOBP17 from *Bradysia odoriphaga* (Zhao et al., 2018), MsepOBP19 from *Mythimna separata* (Chang et al., 2017), AmalOBP8 from *Agrilus mali* (Cui et al., 2018), and AlucOBP6 from *Apolygus lucorum* (Hua et al., 2012). The chemoreception sensilla have been found on the wings of *A. mali*, as well as the taste organ and taste bristles and were also located on the wings of *Drosophila melanogaster* (Galindo and Smith, 2014). We speculated that the GmolOBP7 may play an important role in olfactory or gustatory perception, and further studies with non-volatile secondary metabolites of host plants are needed to verify this.

*G. molesta* thrives mainly on plants of the rosaceae family, and the peach and pear are considered the optimal host plants (Rice et al., 1972; Rajapakse et al., 2006). Plant volatiles serve as olfactory cues for *G. molesta* orientation, and guide the adults to switch from peach orchards to pear orchards during the growing season (Zhao et al., 1989; Najar-Rodriguez et al., 2013). We selected four sex pheromone components and 31 potential host-plant volatiles or its analogs, to determine the binding characteristics of rGmolOBP7. The sex pheromone components have been identified and widely used in the sexual trapping of male *G. molesta* (Cardé et al., 1975, 1979; Reinke et al., 2014). The tested volatiles are known to be emitted from peach shoots and pear fruits. EAG studies on *G. molesta* have shown that some saturated and unsaturated volatile components of aldehydes, alcohols, acetate esters, terpenes and benzonitriles can effectively elicit responses from the antennal lobes of adult moths (Natale et al., 2003; Piñero and Dorn, 2009; Lu et al., 2015). Behavior response assays also indicated that the individual volatile component or mixture of several volatile compounds caused obvious attraction in adult moths (Natale et al., 2004; Piñero et al., 2008; Il'ichev et al., 2009; Yu et al., 2015).

The binding assays showed that GmolOBP7 has broad binding activities to various ligands including aldehydes, alcohols, esters, terpenoids and nitriles compounds. Pear ester, lauraldehyde, and dodecanol were the first three strongest ligands that bound to rGmolOBP7. Previous reports confirmed that the minor sex pheromone component 12:OH only elicited a weak EAG response to male antennae of *G.moletsa*. Its main function is a synergist attractant, that increases the frequency of male landing and is a stimulus that induces mating behavior when the male and female are close to each other, or when the male is close to pheromone lures (Cardé et al., 1975, 1979). EAG responses of GmolOBP7-dsRNA-treated males to 12:OH were significantly reduced compared with GFP-dsRNA-injected and non-injected controls. The simplest explanation is that GmolOBP7 may be involved in the perception of the sex pheromone 12:OH, and a behavioral response test of GmolOBP7-daRNA-treated to 12:OH is required to confirm this in future studies. Pear ester (Ethyl (*E*,*Z*)-2,4-decadienoate) belongs to a volatile derived from pear fruits and is widely applied in trapping female codling moth, *Cydia pomonella*, which is a closely related species of *G. molesta* (Vanessa et al., 2008). Pear ester exhibited the strongest binding affinity with GmolOBP7, and the EAG response values of dsRNA-treated males and females to pear ester were significantly decreased. GmolOBP7 may play the same role in perception of pear ester in male and female moths. rGmolOBP7 showed strong binding ability to lauraldehyde, but the EAG responses of dsRNA-treated male and female moths, to this compound, were not significantly different compared to non-injected controls. OBPs have a binding pocket formed by a six-α-helix fold, and usually have similar binding affinities to the ligand with the same structure and size. For example, *Locusta migratoria* LmigOBP1 binds to pentadecanol (C15), 2-pentadecanone (C15) and ethyl tridecanoate (C15) (Jiang et al., 2009), *Bombyx mori* BmorGOBP2 binds to (10*E*,12*Z*)-hexadecadien-1-ol (bombykol) and (10*E*,12*Z*)-hexadecadienal (bombykal) (Zhou et al., 2009), *Loxostege sticticalis* LstiGOBP2 binds to 1-hexanol and 1-hexanal (Yin et al., 2012). We speculated that GmolOBP7 bound to lauraldehyde because of its size. Similar to pear ester and 12:OH, the lauraldehyde is also a derivative of a linear aliphatic hydrocarbon with 12 carbon atoms in the main chain. The molecular size of these three compounds are similar.

The binding assays were performed as recombinant OBPs expressed *in vitro* and the binding of OBPs with the ligands are affected by the shape and amino acid residues of the binding pocket of proteins, as well as the carbon-chain lengths, functional groups, isomers, and C = C bonds of ligands (Sandler et al., 2000; Mohanty et al., 2004; Wogulis et al., 2006; Li et al., 2008; Christina et al., 2017). OBPs may bind to many tested ligands with similar structures or sizes. Whether the odorants with strong binding activity to OBPs play a role in chemoreception such as mating and host selecting in insects, still needs to be verified by electrophysiological and behavioral assays. The methods of the RNAi combined with an EAG assay is an effective way to verify whether the binding-active odorants can be recognized by insects (Zhang et al., 2017). We found that GmolOBP7 exhibited binding activities in 21 of 35 tested ligands. The EAG assays preliminary revealed that GmolOBP7 may be involved in the detection of 12:OH and pear ester, however, whether GmolOBP7 participates in the perception of other remaining binding-active odorants, requires further functional verification.

## Author Contributions

X-LC, G-WL, X-LX, and J-XW conceived and designed the experimental plan. X-LC and G-WL performed the experiments. X-LC, J-XW, and X-LX analyzed and processed the data. X-LC wrote the paper. All authors read and agreed to publish this paper.

### Conflict of Interest Statement

The authors declare that the research was conducted in the absence of any commercial or financial relationships that could be construed as a potential conflict of interest.

## Supplementary Material

Figure S1 Nucleotide sequence and deduced aa sequence of GmolOBP7 in *Grapholita molesta*. The initiation and termination codons are indicated in boxes. The six conserved cysteines are marked by a circle with a blue background.

Figure S2 Standard curves and melting curves of reference and target genes in qRT-PCR. **(A,C,E)** represents the standard curves of *Gmol*β*-actin, GmolEF1*-α, and *GmolOBP*7, respectively. **(B,D,F)** were the melting curves of *Gmol*β*-actin*, and *GmolEF1*-α, and *GmolOBP*7, respectively.
