# Supplementary figures and images for: Molecular and Functional Characterization of Odorant Binding Protein 7 From the Oriental Fruit Moth Grapholita molesta (Busck) (Lepidoptera: Tortricidae)
Source: Front Physiol. 2018 Dec 10;9:1762. doi: 10.3389/fphys.2018.01762 (PMC6295574; doi:10.3389/fphys.2018.01762)

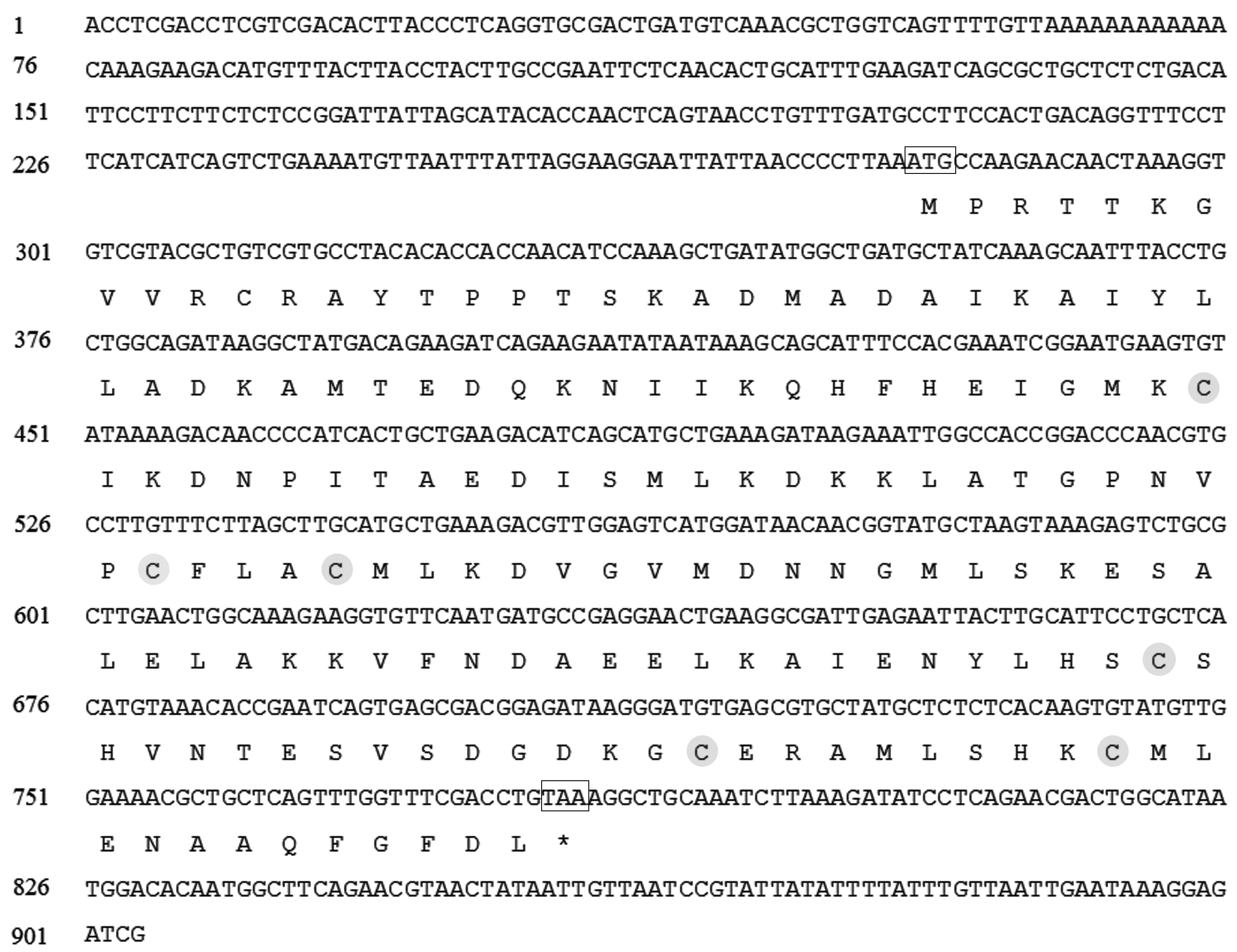

Supplement: Figure S1 — Nucleotide sequence and deduced aa sequence of GmolOBP7 in Grapholita molesta. The initiation and termination codons are indicated in boxes. The six conserved cysteines are marked by a circle with a blue background. [file Image_1.TIF]

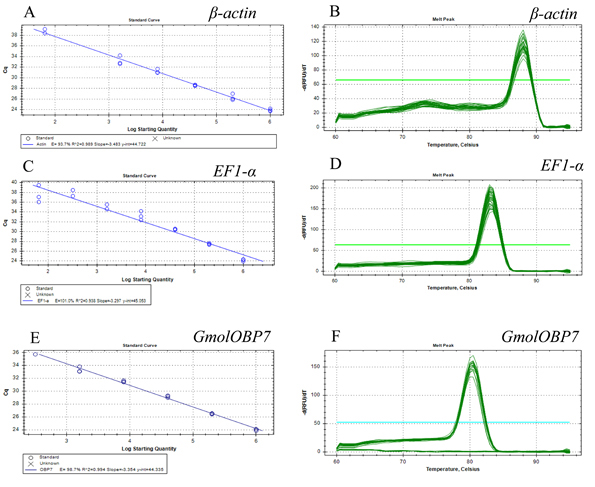

Supplement: Figure S2 — Standard curves and melting curves of reference and target genes in qRT-PCR. (A,C,E) represents the standard curves of Gmolβ-actin, GmolEF1-α, and GmolOBP7, respectively. (B,D,F) were the melting curves of Gmolβ-actin, and GmolEF1-α, and GmolOBP7, respectively. [file Image_2.JPEG]
